# Early bacterial spreading and inflammatory profile in a pneumosepsis model

**DOI:** 10.1186/cc14042

**Published:** 2014-12-03

**Authors:** M de Carvalho Gonçalves, V Horewicz, J Assreuy

**Affiliations:** 1 Department of Pharmacology, Universidade Federal de Santa Catarina, Florianopolis, Brazil

## Introduction

Pneumonia is the major cause of sepsis, responsible for almost one-half of all sources of infection [1]. Sepsis and septic shock lead to organ failure and death. Spreading of microorganisms and their toxins through the blood could contribute to the organ dysfunction. The heart, liver and kidney are examples of organs damaged during the systemic infection and organ failure predicts poor prognosis in patients with sepsis [2]. However the correlation, if any, between bacterial spreading and organ injury is unclear. Thus, the aim of this study was to study the bacterial systemic spreading from a localized infection along with time and the target organ inflammatory profile using proinflammatory cytokines as surrogate markers.

## Methods

Pneumosepsis was induced by *Klebsiella pneumoniae *as described in [3]. The number of viable bacteria inoculated was 10^9 ^colony-forming units to achieve a mortality rate of ~50% by 48 hours. Blood, lung, heart, liver, spleen, kidney and brain were aseptically harvested, homogenized and plated on Müeller-Hinton agar dishes, for 18 hours at 37°C. Plasma and the tissues homogenates were assayed for interleukin-6 (IL-6) and interleukin-1β (IL-1β) using commercially available enzyme-linked immunosorbent assay kits according to the manufacturer's recommendations (PeproTech Inc., Rocky Hills, NJ, USA).

## Results

Lung inoculation with *K. pneumoniae *evolved to systemic spreading of bacteria to all organs, mostly the liver and kidney. Surprisingly, bacteria were found as early as 30 minutes in vital organs such as the brain and heart. The infection in the organs rose steadily up to 24 to 48 hours. Significant increases in IL-6 and IL-1β were found in the plasma, 24 hours after infection. However, cytokine levels in the organs were as high as fivefold the plasma levels.

## Conclusion

Our data show that the early bacterial dissemination may be important for the onset of organ inflammation. Assuming that the higher organ cytokine level is a marker of an ongoing inflammation, this may explain organ dysfunction during sepsis. Thus, our study suggests that systemic (meaning blood) parameters may not reflect the severity of inflammation/dysfunction in target organs, and that might be the determinant to sepsis outcome.
